# Development and validation of a clinical-ready nomogram for predicting postoperative pulmonary infection after coronary artery bypass grafting

**DOI:** 10.3389/fcvm.2026.1880977

**Published:** 2026-09-08

**Authors:** Yangyao Peng, Bangyu Guo, Jingjing Huang, Yuan Chen, Yanrong Wu, Qian Hu, Caixia Gao, Fen Hu

**Affiliations:** 1 Department of Cardiovascular Surgery, Zhongnan Hospital of Wuhan University, Wuhan, China; 2 Hubei Provincial Engineering Research Center of Minimally Invasive Cardiovascular Surgery, Wuhan, China; 3 Wuhan Clinical Research Center for Minimally Invasive Treatment of Structural Heart Disease, Wuhan, China; 4 Department of Plastic Surgery, Zhongnan Hospital of Wuhan University, Wuhan, China; 5 Department of Cardiac Surgery, Tongji Medical College, Union Hospital, Huazhong University of Science and Technology, Wuhan, China; 6 Department of Nursing, Zhongnan Hospital of Wuhan University, Wuhan, China; 7 Wuhan University Center for Critical Care and Anesthesia Nursing Research, Wuhan, China

**Keywords:** coronary artery bypass grafting, prediction model, pulmonary infection, real world accident data, risk factors

## Abstract

**Background:**

Postoperative pulmonary infection (PPI) remains a significant complication following coronary artery bypass grafting (CABG). This study aimed to identify key risk factors and develop a predictive model to facilitate early risk stratification and individualized intervention strategies.

**Method:**

We retrospectively reviewed data from 477 patients who underwent CABG at Zhongnan Hospital of Wuhan University between January, 2020, and December, 2025. Patients were randomly assigned to a training cohort (*n* = 334) and a validation cohort (*n* = 143) in a 7:3 ratio. A combination of Boruta random forest algorithm and Least Absolute Shrinkage and Selection Operator (LASSO) regression was used to select optimal predictors. Model performance was evaluated using the area under the receiver operating characteristic curve (AUC) for discrimination, the Hosmer–Lemeshow test and calibration curves for calibration, and decision curve analysis (DCA) for clinical utility.

**Result:**

Six variables were identified as independent predictors: tracheal intubation >24 h, operative duration >10 h, concomitant surgery, intraoperative red blood cell (RBC) transfusion, intraoperative blood loss, and use of intra-aortic balloon pump (IABP). The model demonstrated good discrimination with an AUC of 0·742 (95% CI 0·668–0·817) in the training cohort and 0·745 (95% CI 0·637–0·853) in the validation cohort. Calibration was excellent in both cohorts (training: *χ*^2^ = 6·179, *P* = 0·630; validation: *χ*^2^ = 4·894, *P* = 0·770), and DCA indicated significant clinical net benefit across a wide range of threshold probabilities.

**Conclusion:**

The developed nomogram, based on six readily available clinical variables, provides a robust and intuitive tool for identifying patients at high risk of PPI after CABG. Early quantification of risk may assist clinicians in optimizing perioperative management.

## 1 Introduction

Coronary heart disease (CHD), also known as ischaemic heart disease, is characterised by atherosclerosis within the coronary arteries and their principal branches. This vascular narrowing or occlusion leads to insufficient cardiac perfusion, subsequently precipitating myocardial ischaemia, hypoxia, or even necrotic damage (1). Driven by rapid socioeconomic development, improved living standards, and the growing severity of population ageing associated with prolonged life expectancy, the incidence of CHD has risen in recent years and is projected to maintain a rapid upward trajectory (2, 3). As a major public health challenge, CHD is epidemiologically defined by high morbidity and mortality. Beyond the severe impairment of patients’ physical and psychological wellbeing, the disease imposes a substantial burden on both families and society (4, 5). Coronary artery bypass grafting (CABG) has emerged as a reliable and preferred therapeutic option for patients refractory to pharmacological therapies and stent interventions (6). Recognized as a crucial modality in the management of CHD, CABG utilises autologous vessels to establish novel perfusion conduits between the proximal and distal segments of the coronary stenosis. This approach successfully bypasses the occlusion, directly restoring adequate blood supply to the myocardium (7, 8).

Despite the progressive refinement of coronary artery bypass grafting (CABG) techniques, the incidence of postoperative complications remains a non-negligible concern. As a major surgical intervention requiring thoracotomy, CABG inevitably imposes significant stress on the respiratory system, typically resulting in a precipitous decline in pulmonary function during the early postoperative period—generally within the first week (9, 10). Against this backdrop of impaired host defences, pulmonary infection has emerged as one of the most frequent and severe complications following CABG, substantially compromising the quality of postoperative recovery (11) and potentially serving as a decisive factor limiting long-term survival (12). Failure to promptly address such infections not only exacerbates the financial burden on patients and impedes clinical convalescence but also significantly heightens the risk of mortality.

Consequently, this remains an urgent clinical challenge. Notably, there is currently a lack of precision assessment tools specifically designed to evaluate the risk of pulmonary infection within this specific cohort.

## 2 Materials and methods

### 2.1 Study population

A retrospective cohort comprising 477 patients who underwent CABG at the Department of Cardiovascular Surgery, Zhongnan Hospital of Wuhan University, between January, 2020, and December, 2025, was included in this study. The study protocol received ethical approval from the Ethics Committee of Zhongnan Hospital of Wuhan University (approval No. 2025017K).

### 2.2 Data collection

A comprehensive dataset consisting of 36 clinical variables was extracted for each patient. These variables encompassed demographic characteristics, past medical history, valvular function, cardiopulmonary bypass parameters, intraoperative pharmacotherapy, and fluid balance (intake and output). The clinical diagnosis of pulmonary infection was established based on the presence of new or progressive infiltrates, consolidation, or ground-glass opacities on chest radiography or computed tomography (CT), concomitant with at least two of the following three clinical criteria: (1) fever (body temperature >38 °C); (2) purulent airway secretions; and (3) an abnormal leucocyte count, defined as either leucocytosis (>10 × 10^9^/L) or leucopenia (<4 × 10^9^/L).

### 2.3 Statistical analysis

Prior to statistical modeling, the dataset was rigorously screened. Outliers and missing data were appropriately addressed using the multiple imputation method to minimize bias and preserve statistical power. Data processing and statistical analyses were conducted using R (version 4.3.2) and SPSS (version 27.0). Continuous variables conforming to a normal distribution were summarised as mean ± standard deviation (x¯ ± s), with inter-group comparisons performed using the independent samples t-test. Non-normally distributed continuous data were expressed as median and interquartile range [M (P25, P75)] and analysed via the Mann–Whitney U test. Categorical variables were reported as frequencies and percentages (%), and the *χ*^2^ test was applied to assess differences between groups.

To optimise variable selection, a dual machine-learning approach was employed. Initially, least absolute shrinkage and selection operator (LASSO) regression, incorporating 10-fold cross-validation, was utilised to identify candidate variables with non-zero coefficients. Concurrently, the Boruta algorithm, a random-forest-based wrapper method, was applied for all-relevant feature selection to isolate statistically significant predictors. The intersection of the variables identified by these two independent algorithms constituted the final set of predictors. These predictors were subsequently incorporated into a multivariable logistic regression analysis to construct the prediction model, which was visualised as a nomogram. Model discrimination was quantified using the area under the receiver operating characteristic (ROC) curve (AUC). Calibration was assessed via the Hosmer-Lemeshow (H-L) test alongside calibration curves, while decision curve analysis (DCA) was implemented to evaluate the clinical utility of the model.

## 3 Result

### 3.1 Baseline characteristics

**Figure 1:**
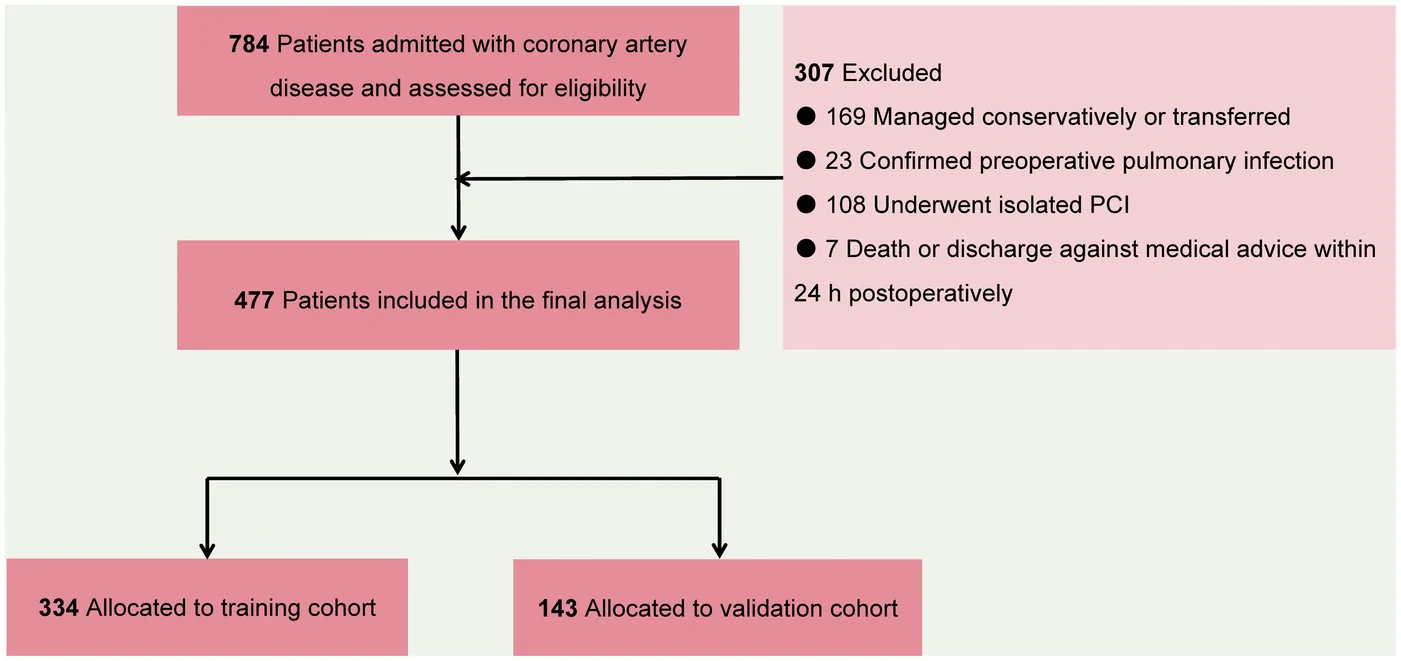
Flowchart of the process of patient enrollment.

A total of 784 patients admitted with coronary artery disease were initially assessed for eligibility. After excluding patients based on predefined criteria—including 169 managed conservatively, 23 with confirmed preoperative pulmonary infection, 108 who underwent isolated PCI, and 7 who died or were discharged against medical advice within 24 h postoperatively—477 patients were ultimately included in the final analysis (Figure 1). The overall incidence of postoperative pulmonary infection was 17.2% (82/477). Using a random computer-generated sequence (7:3 ratio), the study population was divided into a training cohort (*n* = 334) and a validation cohort (*n* = 143). Postoperative pulmonary infection occurred in 58 patients (17.4%) in the training cohort and 24 patients (16.8%) in the validation cohort, demonstrating a balanced event distribution. Detailed baseline characteristics of the training cohort are summarized in Table 1.

**Table 1:** Baseline characteristics of patients in the training cohort.

Variables	Total (*n* = 334)	Non-pneumonia group (*n* = 276)	Pneumonia group (*n* = 58)	*χ*²/Z	*P*
Male, *n*(%)	253 (75.75)	204 (73.91)	49 (84.48)	χ² = 2.91	0.088
Age (years)	61.00 (55.00, 68.00)	61.00 (55.00, 67.00)	62.00 (57.00, 68.75)	Z = −1.01	0.312
Smoking, *n*(%)	83 (24.85)	71 (25.72)	12 (20.69)	χ² = 0.65	0.420
Alcohol consumption, *n*(%)	73 (21.86)	63 (22.83)	10 (17.24)	χ² = 0.88	0.350
Hypertension, *n*(%)	209 (62.57)	176 (63.77)	33 (56.90)	χ² = 0.97	0.326
Diabetes, *n*(%)	106 (31.74)	85 (30.80)	21 (36.21)	χ² = 0.65	0.421
Stroke, *n*(%)	22 (6.59)	18 (6.52)	4 (6.90)	χ² = 0.00	1.000
COPD, *n*(%)	3 (0.90)	3 (1.09)	0 (0.00)	-	1.000
Renal insufficiency, *n*(%)	55 (16.47)	38 (13.77)	17 (29.31)	χ² = 8.42	0.004
Concomitant valvular disease, *n*(%)	98 (29.34)	76 (27.54)	22 (37.93)	χ² = 2.50	0.114
Concomitant mitral valve disease, *n*(%)	71 (21.26)	53 (19.20)	18 (31.03)	χ² = 4.01	0.045
Concomitant tricuspid valve disease, *n*(%)	34 (10.18)	25 (9.06)	9 (15.52)	χ² = 2.19	0.139
Concomitant aortic valve disease, *n*(%)	49 (14.67)	39 (14.13)	10 (17.24)	χ² = 0.37	0.543
IABP support, *n*(%)	49 (14.67)	31 (11.23)	18 (31.03)	χ² = 15.01	<.001
ECMO support, *n*(%)	5 (1.50)	4 (1.45)	1 (1.72)	-	1.000
Intubation time >24 h, *n*(%)	128 (38.32)	88 (31.88)	40 (68.97)	χ² = 27.88	<.001
Intraoperative dopamine use, *n*(%)	284 (85.03)	231 (83.70)	53 (91.38)	χ² = 2.22	0.136
Intraoperative epinephrine use, *n*(%)	187 (55.99)	147 (53.26)	40 (68.97)	χ² = 4.80	0.029
Intraoperative norepinephrine use, *n*(%)	179 (53.59)	145 (52.54)	34 (58.62)	χ² = 0.71	0.398
Intraoperative blood loss >1,000 mL, *n*(%)	61 (18.26)	43 (15.58)	18 (31.03)	χ² = 7.67	0.006
Preoperative LOS >7 days, *n*(%)	112 (33.53)	86 (31.16)	26 (44.83)	χ² = 4.02	0.045
Operative duration >10 h, *n*(%)	85 (25.45)	64 (23.19)	21 (36.21)	χ² = 4.28	0.039
Concomitant surgery, *n*(%)^a^	115 (34.43)	83 (30.07)	32 (55.17)	χ² = 13.37	<.001
Abnormal BMI, *n*(%)	185 (55.39)	158 (57.25)	27 (46.55)	χ² = 2.22	0.136
Cardiopulmonary bypass surgery, *n*(%)^b^	110 (32.93)	84 (30.43)	26 (44.83)	χ² = 4.50	0.034
Operative duration (min)	520.00 (450.00, 600.00)	502.50 (443.75, 600.00)	550.00 (510.00, 613.75)	Z = −3.02	0.002
Preoperative LOS (days)	11.00 (8.00, 15.00)	11.00 (8.00, 15.00)	12.50 (8.00, 18.75)	Z = −1.42	0.156
Heparin-protamine time (min)	170.05 (127.17, 229.55)	166.55 (121.97, 224.71)	187.58 (144.22, 252.32)	Z = −1.85	0.065
Intraoperative urine output (mL)	1,300.00 (762.50, 1,800.00)	1,200.00 (700.00, 1,800.00)	1,400.00 (925.00, 2,000.00)	Z = −1.10	0.269
Intraoperative blood loss (mL)	500.00 (300.00, 800.00)	500.00 (300.00, 800.00)	600.00 (400.00, 1,150.00)	Z = −2.35	0.019
Intraoperative colloids (mL)	1,000.00 (500.00, 1,000.00)	1,000.00 (500.00, 1,000.00)	1,000.00 (500.00, 1,000.00)	Z = −0.01	0.995
Intraoperative RBC transfusion (U)	0.00 (0.00, 2.00)	0.00 (0.00, 2.00)	2.00 (0.00, 3.38)	Z = −3.75	<.001
Intraoperative crystalloids (mL)	1,000.00 (500.00, 1,500.00)	1,000.00 (500.00, 1,500.00)	1,000.00 (525.00, 1,500.00)	Z = −0.43	0.670
Intraoperative autologous transfusion (mL)	250.00 (200.00, 500.00)	250.00 (187.50, 500.00)	350.00 (250.00, 500.00)	Z = −1.00	0.316
Intraoperative plasma transfusion (U)	0.00 (0.00, 350.00)	0.00 (0.00, 300.00)	300.00 (0.00, 487.50)	Z = −3.28	0.001

Z: Mann–Whitney test, χ²: Chi-square test, -: Fisher exact.

M, Median; Q₁, 1st Quartile; Q₃, 3rd Quartile.

a Concomitant surgeries primarily included valvular surgeries, aortic dissection surgeries, and other major cardiac procedures.

b The remaining 224 patients (67.07%) who did not undergo cardiopulmonary bypass surgery were treated with off-pump CABG (OPCAB).

### 3.2 Feature selection

**Figure 2:**
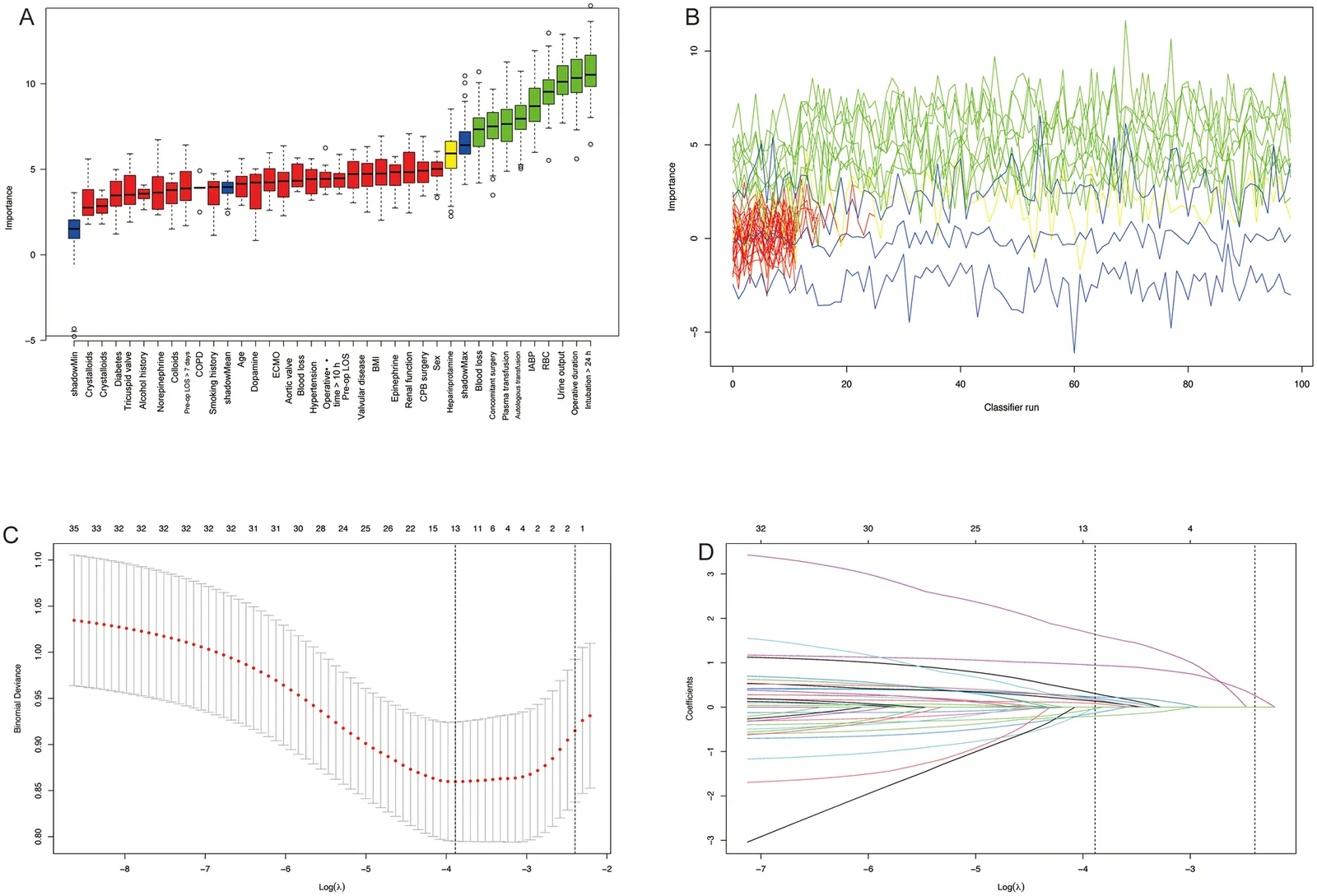
Variable selection using the Boruta algorithm and LASSO regression. (**A**) Variable importance boxplots generated by the Boruta algorithm. (**B**) Tracking of feature importance over classifier runs. (**C**) Selection of the optimal penalization parameter in the LASSO model using cross-validation. (**D**) LASSO coefficient profiles of the candidate variables.

All candidate factors were evaluated using the Boruta algorithm and LASSO regression to screen for variables associated with pulmonary infection in patients undergoing CABG. The Boruta algorithm identified nine crucial features: use of an intra-aortic balloon pump (IABP), duration of tracheal intubation >24 h, concomitant surgery, operative duration, intraoperative urine output, intraoperative blood loss, and intraoperative transfusion of red blood cells, autologous blood, and plasma (Figures 2A,B). LASSO regression, applying the minimum 10-fold cross-validation error (lambda.min, *λ*=0.02) as the optimal threshold, selected 13 predictive factors with non-zero coefficients: sex, history of alcohol consumption, history of hypertension, use of IABP, use of extracorporeal membrane oxygenation (ECMO), duration of tracheal intubation >24 h, renal dysfunction, preoperative hospital stay >7 days, operative duration >10 h, concomitant surgery, intraoperative blood loss, and intraoperative red blood cell transfusion, and intraoperative epinephrine use (Figures 2C,D). Based on the intersection of the results from both algorithms, five predictive factors were initially determined. Concurrently, we observed that “operative duration” (a continuous variable) and “operative duration >10 h” (a categorical variable) were independently selected by the two methods, representing the same clinical parameter in different data formats. To facilitate the construction of a user-friendly predictive model, the dichotomous variable “operative duration >10 h” was retained. Ultimately, a total of six variables were entered into the multivariable logistic regression model. To ensure complete harmony with the visualization in the final nomogram, these predictors were explicitly formatted as follows: four were treated as categorical variables (use of IABP, duration of tracheal intubation >24 h, concomitant surgery, and operative duration >10 h), and two were treated as continuous variables (intraoperative blood loss [mL] and intraoperative RBC transfusion [U]).

### 3.3 Model construction and validation

**Figure 3:**
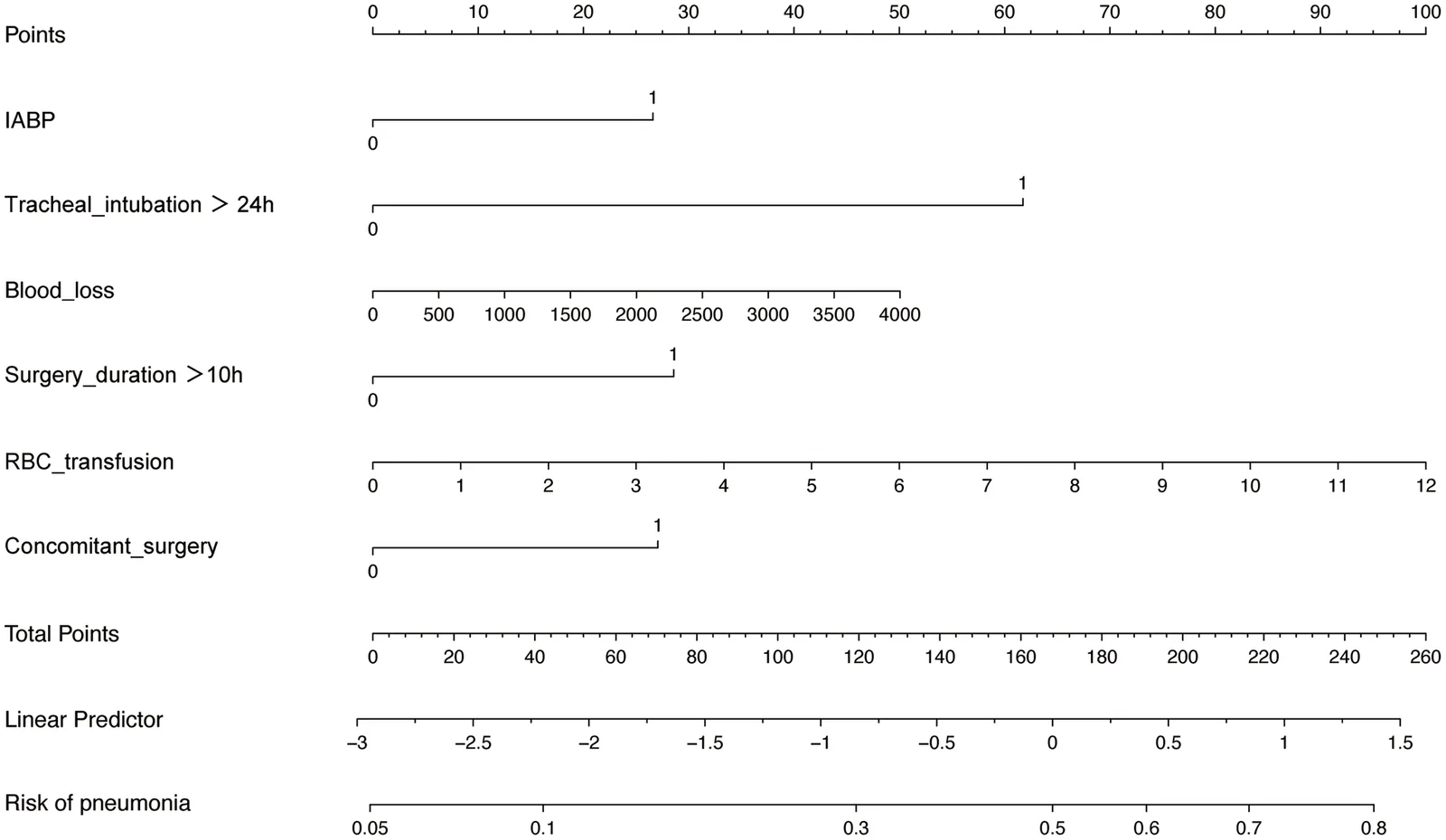
Nomogram for predicting postoperative pulmonary infection following CABG.

A nomogram was constructed based on the analysis of the selected predictors. As illustrated in Figure 3, a total score of 165 corresponds to a probability of pulmonary infection exceeding 50%, whereas a total score of 245 signifies a substantially augmented risk of developing a pulmonary infection. The detailed parameters of the full multivariable logistic regression model, including the intercept, coefficients, and variable coding, are provided in Supplementary Table S1.

**Figure 4:**
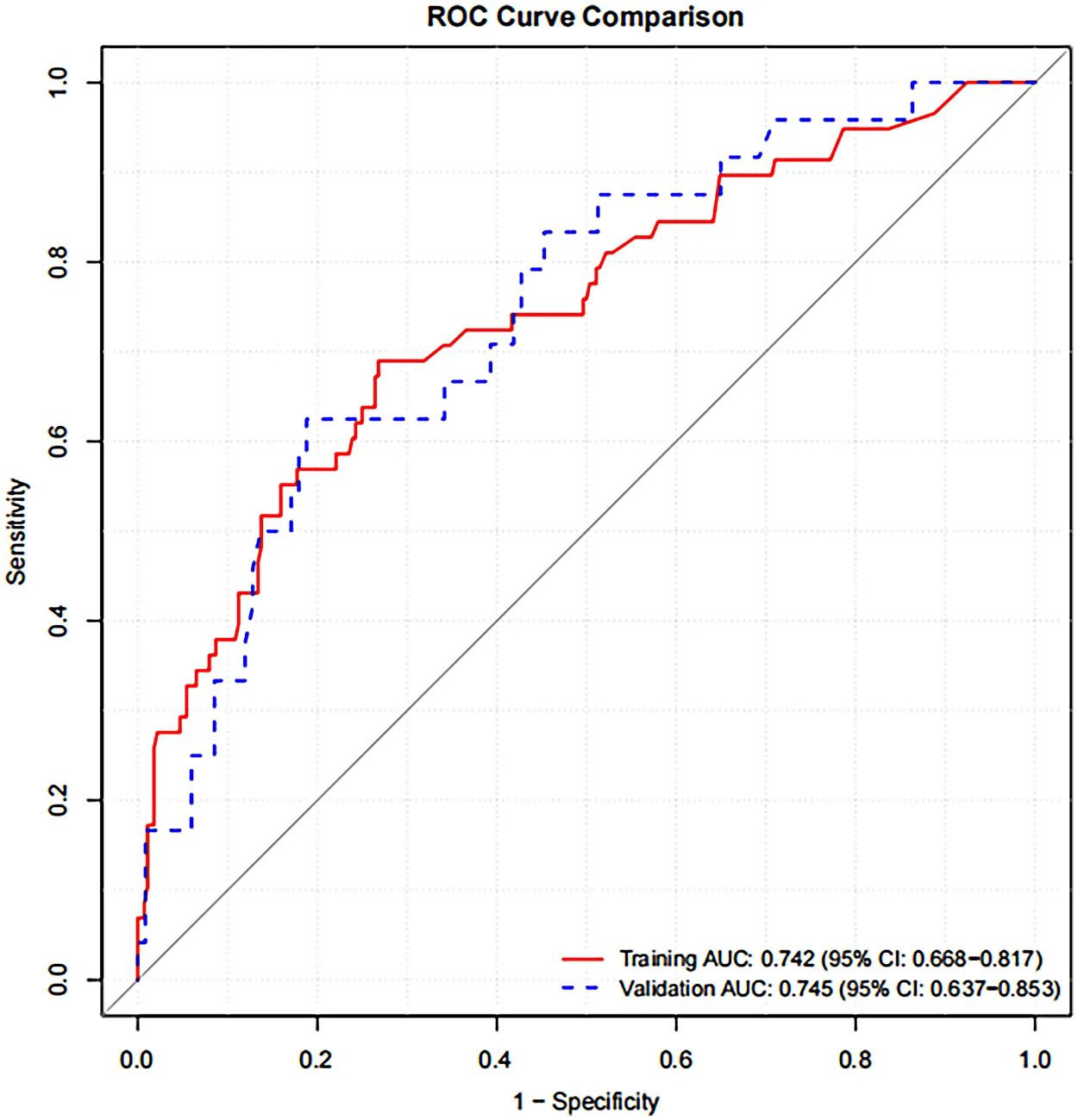
ROC curve comparison.

**Figure 5:**
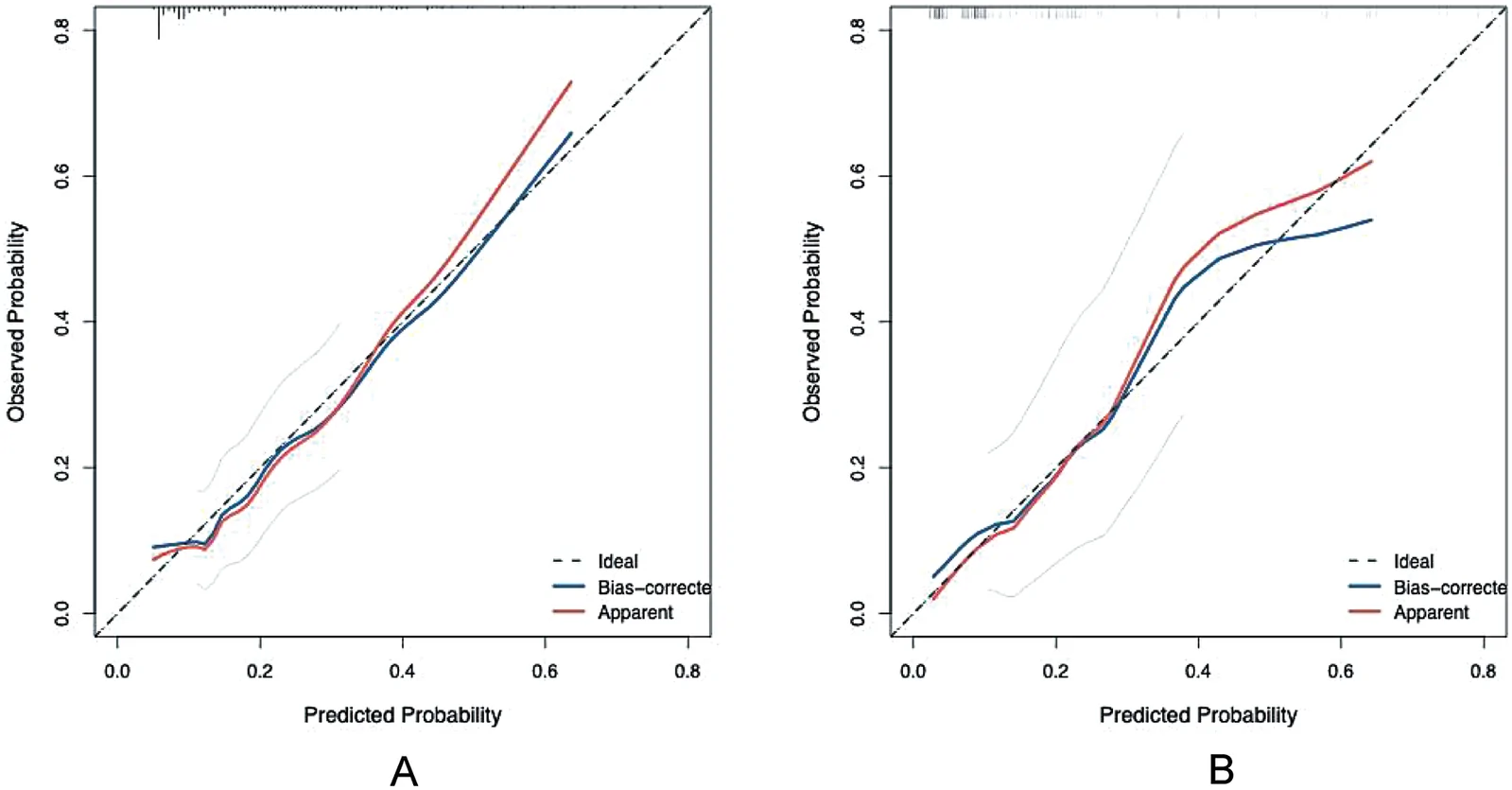
Calibration curves of the prediction model for postoperative pulmonary infection following CABG (**A**: training cohort; **B**: test cohort).

**Figure 6:**
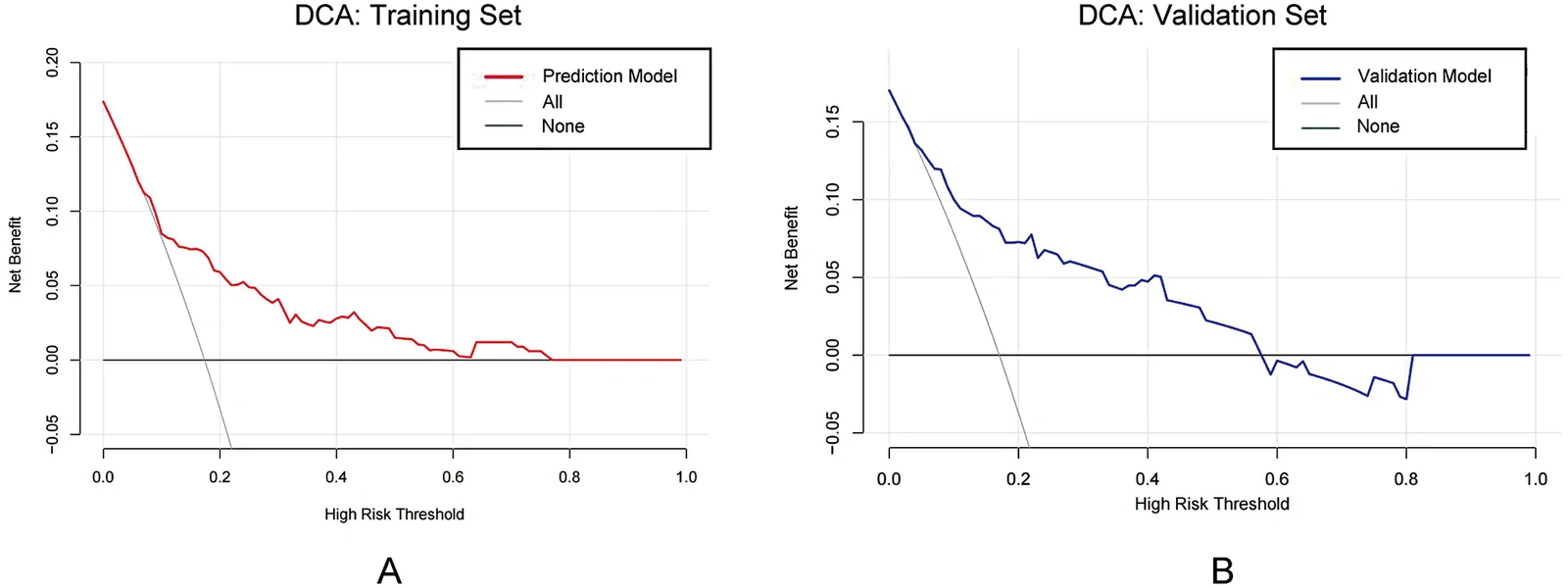
Decision curves of the prediction model for postoperative pulmonary infection following CABG (**A**: training cohort; **B**: test cohort).

The constructed prediction model was comprehensively evaluated using multiple metrics. In terms of discrimination, the area under the receiver operating characteristic curve (AUC) was 0.742 (95% CI 0.668–0.817) in the training cohort and 0.745 (95% CI 0.637–0.853) in the validation cohort, indicating that the model possessed robust discriminative ability (Figure 4). Regarding calibration, the Hosmer-Lemeshow goodness-of-fit test revealed no significant deviation in either the training cohort (*χ*^2^ = 6.179, *p* = 0.630) or the validation cohort (*χ*^2^ = 4.894, *p* = 0.770). Furthermore, the calibration curves closely approximated the ideal reference lines (Figure 5), suggesting a high degree of concordance between the predicted probabilities and the actual risk of infection. Finally, the clinical utility of the model was assessed via decision curve analysis (DCA). The results demonstrated that the model yielded a substantial net benefit across a broad range of threshold probabilities, affirming its pragmatic value in clinical settings (Figure 6).

## 4 Discussion

Despite the definitive long-term efficacy of CABG, postoperative pulmonary infection remains a critical barrier to early recovery. Consequently, the development of a precise risk-prediction model to identify high-risk populations at an early stage holds substantial clinical value for tailoring individualised interventional strategies and optimising the allocation of health-care resources.

Prolonged operative duration, coupled with the concurrent execution of additional surgical procedures, engenders a state of profound physiological stress characteristic of complex surgical environments. Extended surgical interventions invariably necessitate prolonged exposure of mediastinal and thoracic tissues to the external environment, thereby significantly increasing the propensity for pathogen deposition and colonisation (13). From the perspective of physiological stress, an extended operative duration is correlated with elevated systemic secretion of glucocorticoids and catecholamines. This endogenous stress response triggers a transient yet severe immunosuppression, compromising the defensive capabilities of alveolar macrophages (14). Furthermore, the establishment of an artificial airway entirely bypasses the physiological warming, humidification, and filtration functions of the upper respiratory tract, leaving the lower respiratory tract directly exposed to the nosocomial environment. As the duration of intubation increases, the impairment of the mucociliary clearance system of the airway mucosa progressively worsens. This deterioration results in inadequate drainage of secretions and fosters the formation of biofilms at the distal tip of the endotracheal tube (15). Additionally, the continuous administration of sedative and analgesic agents requisite for prolonged mechanical ventilation further suppresses the patient's spontaneous cough reflex, thereby substantially elevating the risk of silent aspiration.

The mechanistic link between intraoperative red blood cell (RBC) transfusion and pulmonary infection is primarily underpinned by transfusion-related immunomodulation (TRIM). The infusion of allogeneic RBCs induces a cascade of immunotolerance responses within the recipient, leading to the dysregulation of T-cell subsets and the suppression of natural killer cell activity (16, 17). Furthermore, in concordance with previous findings (18, 19), the intraoperative transfusion of RBCs is associated with an elevated incidence of postoperative pneumonia. The dose-dependent TRIM mediated by exposure to allogeneic blood, coupled with potential microcirculatory volume overload, synergistically deteriorates the pulmonary microenvironment. Although the fundamental rationale for blood transfusion is to ameliorate tissue hypoxia by augmenting haemoglobin concentrations, this theoretical oxygen-carrying benefit is entirely counteracted by the consequent compromise of the immune barrier and the exacerbation of pulmonary interstitial oedema associated with the administration of exogenous RBCs.

The disruption of the pulmonary defensive microenvironment induced by intraoperative blood loss is multidimensional. Acute haemorrhage directly precipitates the rapid depletion of core humoral and cellular immune components, including circulating leucocytes, complement proteins, and immunoglobulins, thereby plunging the patient into a state of immunosuppression during the early postoperative phase. Concurrently, the precipitous decline in effective circulating volume induces transient pulmonary hypoperfusion; during subsequent haemodynamic restoration and fluid resuscitation, this process frequently triggers pulmonary ischaemia-reperfusion injury (20). The massive release of oxygen free radicals and pro-inflammatory cytokines directly compromises the alveolar-capillary barrier, culminating in an abnormal escalation of vascular endothelial permeability. Furthermore, the compensatory fluid resuscitation requisite for maintaining perioperative haemodynamic stability inevitably results in haemodilution and a profound reduction in plasma colloid osmotic pressure, thereby precipitating pulmonary interstitial exudation and oedema (21). These pathological alterations, fundamentally characterised by pulmonary exudation and oedema, not only impair the mucociliary clearance system of the airway epithelium but also cultivate an optimal pathological microenvironment for the colonisation and extensive proliferation of pathogenic bacteria within the lower respiratory tract (22).

IABP is predominantly utilised in high-risk patients presenting with severe left ventricular dysfunction, cardiogenic shock, or low cardiac output syndrome; as such, its deployment is intrinsically a hallmark of severely compromised cardiac reserve (23–25). These patients frequently exhibit elevated left atrial pressure, pulmonary capillary wedge pressure, and mean pulmonary arterial pressure, indicative of profound pulmonary venous hypertension and a state of congestion (26). Although IABP insertion can partially attenuate pulmonary arterial and capillary wedge pressures, the underlying pulmonary congestion often persists (27). This congestive pulmonary environment precipitates interstitial oedema, diminished lung compliance, increased serous airway secretions, and an augmented risk of atelectasis and infection, representing a classic pathophysiological cascade for cardiogenic lung injury and postoperative pulmonary complications (28). In patients with cardiac dysfunction, chronic pulmonary venous hypertension induces interstitial and alveolar oedema and compromises local host defences, thereby creating a susceptible foundation for bacterial colonisation and subsequent pulmonary infection. Concurrently, within traditional nursing paradigms, patients requiring IABP support are frequently confined to a prolonged supine position to prevent catheter displacement. This immobilisation precludes early turning, sitting, and effective postural drainage, predisposing patients to the gravitational pooling of fluid-rich secretions in the basal and dependent pulmonary segments, which further promotes atelectasis and lower respiratory tract infections (9). Consequently, the requirement for an IABP not only reflects the critical severity of the underlying disease but also constitutes a significant complicating factor in postoperative respiratory care that necessitates meticulous clinical attention.

## 5 Limitation

Several limitations of this study should be acknowledged. First, as a single-center retrospective study, inherent selection bias is unavoidable, and the sample size from our single institution remains relatively limited. Second, the proposed nomogram was only validated internally using a data split method. The lack of external validation across multicenter cohorts currently limits the broader generalizability and robustness of the model in diverse healthcare settings or different geographical populations. Third, although a comprehensive set of perioperative variables was analyzed, some potential unmeasured confounders were not included in the model, such as specific intraoperative lung-protective ventilation strategies (e.g., tidal volume and positive end-expiratory pressure), perioperative nutritional status, and variations in surgeon experience. Fourth, the cutoffs for intubation duration (>24 h), operative duration (>10 h), and blood loss (1,000 mL in Table 1) were based on clinical convention rather than optimized in this cohort; blood loss itself was analyzed as a continuous variable throughout. Future prospective, large-scale, and multicenter studies are warranted to externally validate and further refine this prediction model.

## 6 Conclusion

In conclusion, this study successfully developed and internally validated a practical risk prediction model for postoperative pulmonary infection following CABG. The developed nomogram demonstrated favorable discrimination, calibration, and clinical net benefit. As an intuitive and convenient clinical tool, this model can assist clinicians in the early identification of high-risk patients during the perioperative period, facilitating the timely implementation of tailored preventive strategies and ultimately improving postoperative outcomes for patients undergoing CABG.

## Data Availability

The raw data supporting the conclusions of this article will be made available by the authors, without undue reservation.
