# Supplementary material for: Development and validation of a clinical-ready nomogram for predicting postoperative pulmonary infection after coronary artery bypass grafting
Source: Front Cardiovasc Med. 2026 Sep 8;13:1880977. doi: 10.3389/fcvm.2026.1880977 (PMC13593506; doi:10.3389/fcvm.2026.1880977)
Supplement: Supplementary file 1 [file Table1.docx]

**Table S1. Full multivariable logistic regression model for postoperative pulmonary infection after CABG**

| **Predictor** | **Coding** | **β (coefficient)** | **SE** | **OR** | **95% CI** | **P value** |
| --- | --- | --- | --- | --- | --- | --- |
| Intercept | — | -2.9322 | 0.3266 | 0.0533 | 0.0281–0.1011 | <0.001 |
| Use of IABP | 0 = No,  1 = Yes | 0.4648 | 0.3942 | 1.5917 | 0.7350–3.4469 | 0.238 |
| Tracheal intubation duration > 24 h | 0 = No,  1 = Yes | 1.0787 | 0.3692 | 2.9408 | 1.4263–6.0634 | 0.003 |
| Concomitant surgery | 0 = No,  1 = Yes | 0.4728 | 0.3368 | 1.6045 | 0.8292–3.1047 | 0.160 |
| Operative duration > 10 h | 0 = No,  1 = Yes | 0.4991 | 0.3367 | 1.6472 | 0.8514–3.1867 | 0.138 |
| Intraoperative blood loss (mL) | Continuous,  per 100 mL | 0.0219 | 0.0254 | 1.0221 | 0.9725–1.0742 | 0.389 |
| Intraoperative RBC transfusion (U) | Continuous,  per 1 U | 0.1456 | 0.0729 | 1.1567 | 1.0028–1.3343 | 0.046 |

**Note:** IABP = intra-aortic balloon pump; RBC = red blood cell; OR = odds ratio; CI = confidence interval; SE = standard error.
